# Impaired Expression of the Salvador Homolog-1 Gene Is Associated with the Development and Progression of Colorectal Cancer

**DOI:** 10.3390/cancers15245771

**Published:** 2023-12-08

**Authors:** Anna Ewa Kowalczyk, Bartlomiej Emil Krazinski, Aleksandra Piotrowska, Jedrzej Grzegrzolka, Janusz Godlewski, Piotr Dziegiel, Zbigniew Kmiec

**Affiliations:** 1 Department of Human Histology and Embryology, School of Medicine, Collegium Medicum, University of Warmia and Mazury in Olsztyn, 10-082 Olsztyn, Poland; bartlomiej.krazinski@uwm.edu.pl (B.E.K.); janusz.godlewski@uwm.edu.pl (J.G.); 2 Division of Histology and Embryology, Department of Human Morphology and Embryology, Wroclaw Medical University, 50-368 Wroclaw, Poland; aleksandra.piotrowska@umw.edu.pl (A.P.); jedrzej.grzegrzolka@umw.edu.pl (J.G.); piotr.dziegiel@umw.edu.pl (P.D.); 3 Department of Histology, Medical University of Gdansk, 80-210 Gdansk, Poland; zbigniew.kmiec@gumed.edu.pl

**Keywords:** SAV1, colorectal cancer, clinicopathological parameters, Hippo pathway

## Abstract

**Simple Summary:**

The incidence of colorectal cancer (CRC) is dramatically increasing. There is a great need to better understand the mechanisms and factors involved in the development and progression of CRC, as well as to assess their diagnostic and prognostic significance. Deactivation of tumor suppressor genes that regulate many cell processes, such as the cell cycle and apoptosis, is often observed in cancer pathogenesis; therefore, the purpose of our study was to analyze the expression level of the *SAV1* gene, which encodes one of the main components of the Hippo suppressor pathway, and to estimate its prognostic significance in CRC. We showed that *SAV1* mRNA and protein levels in tumor tissues differed significantly from those observed in non-cancerous mucosa, and we were the first to provide evidence that reduced SAV1 protein expression is associated with unfavorable clinicopathological parameters in CRC patients and appears to be involved in the development and progression of CRC.

**Abstract:**

Salvador homolog-1 (SAV1) is a component of the Hippo pathway that regulates tissue growth and homeostasis by affecting diverse cell processes, including apoptosis, cell division, and differentiation. The aberrant expression of Hippo pathway components has been observed in various human cancers. This study aimed to examine the expression level of the *SAV1* gene in colorectal cancer (CRC) and its prognostic value and associations with tumor progression. We obtained matched pairs of tumor tissue and non-cancerous mucosa of the large intestine from 94 CRC patients as well as 40 colon biopsies of healthy subjects collected during screening colonoscopy. The tissue samples and CRC cell lines were quantified for *SAV1* mRNA levels using the quantitative polymerase chain reaction method, while SAV1 protein expression was estimated in the paired tissues of CRC patients using immunohistochemistry. The average level of *SAV1* mRNA was decreased in 93.6% of the tumor tissues compared to the corresponding non-cancerous tissues and biopsies of healthy colon mucosa. A downregulated expression of *SAV1* mRNA was also noted in the CRC cell lines. Although the average SAV1 immunoreactivity was increased in the CRC samples compared to the non-cancerous tissues, a decreased immunoreactivity of the SAV1 protein in the tumor specimens was associated with lymph node involvement and higher TNM disease stage and histological grade. The results of our study suggest that the impaired expression of SAV1 is involved in CRC progression.

## 1. Introduction

The maintenance of tissue homeostasis depends on coordinated cell death, proliferation, and differentiation. A disturbance of this balance can result in the development of cancer, which is characterized by abnormal cell growth, usually facilitated by an inhibition of apoptosis. The Hippo pathway was found to be a signal transduction pathway that plays a crucial role in tissue homeostasis, organ size control, and stem cell functions by regulating cell growth, proliferation, differentiation, and apoptosis. This pathway is evolutionarily conserved and was initially identified and characterized in *Drosophila melanogaster*. The core of the mammalian Hippo pathway is composed of a protein complex that includes kinases MST1 and MST2, large tumor suppressors 1 and 2 (LATS1, LATS2), adaptor proteins SAV1 (Salvador family WW domain containing protein 1), and MOB kinase activator 1A and 1B (MOB1A, MOB1B) [1,2]. The Hippo core complex causes the phosphorylation of the transcriptional coactivators and its main effectors, the YAP (Yes-associated protein) and TAZ (PDZ-binding motif) oncoproteins, and their retention in the cytoplasm, where they undergo proteasomal degradation. When the Hippo pathway is dysregulated and inactive or suppressed, YAP and TAZ translocate to the nucleus, where they induce the transcription of genes involved in cell proliferation, survival, and migration. The dysregulation of this pathway leads to aberrant cell growth and has been associated with many human diseases, including cancers [1,2,3,4].

An impaired expression of the Hippo pathway components has also been observed in colorectal cancer (CRC) [5,6]. Worldwide, CRC ranks third in terms of incidence but second in terms of mortality, with an estimated 1.9 million new cases and 935,000 deaths occurring in 2020, representing about 1 in 10 cancer cases and deaths [7]. A better understanding of the mechanisms and molecular background of CRC development and progression may uncover early-stage disease markers and new targets for cancer therapy.

One of the fundamental components of the Hippo pathway, SAV1 (also known as WW45), is a 45 kDa protein thought to be a tumor suppressor, but its clinical and prognostic significance in CRC is unknown. It is encoded by the *SAV1* gene, whose chromosomal location is 14q22.1. SAV1 consists of two WW domains and one coiled-coil domain in the C-terminal region that are reported in proteins whose major feature is to act as a scaffold [8]. It has been noted that SAV1 also plays an antitumor role, independent of the Hippo pathway [9]. The dysfunction of the *SAV1* gene has been shown to affect developing epithelial tissues and induce characteristics of a precancerous state, such as uncontrolled proliferation, loss of contact inhibition, partial loss of epithelial polarity, and block of terminal differentiation [10]. It has been observed that mice with liver-specific ablation of SAV1 manifest an increased liver size and expansion of hepatic progenitor cells and, eventually, develop hepatomas [11]. Moreover, mice with *SAV1*-deficient small and large intestines were found to exhibit an enlarged crypt structure and an increased polyp formation [12].

Abnormal expression of the *SAV1* gene was reported in some types of human cancers and tumor-derived cell lines such as pancreatic ductal adenocarcinoma [13] and breast [14] and clear cell renal cell [15] carcinoma. Aberrant expression of the *SAV1* gene has also been shown to be involved in the pathogenesis of colorectal cancer [16,17], but, so far, no correlation has been established between the *SAV1* gene expression levels and clinicopathological parameters in CRC patients and their overall survival. Also, the expression levels of the *SAV1* gene in CRC have, so far, been studied only in small groups of patients [16,17], and *SAV1* expression in CRC tissue has not previously been compared with that in the colonic mucosa of healthy individuals. For this reason, the purpose of our study was to investigate and compare *SAV1* gene expression in cancerous and non-cancerous colorectal tissue samples from CRC patients, as well as in colorectal mucosal biopsies from healthy individuals. In addition, we examined *SAV1* mRNA levels in CRC cell lines. To assess the prognostic value of SAV1 expression levels, we examined the association of SAV1 expression with clinicopathological parameters, as well as with the overall survival of patients with CRC. We found that impaired *SAV1* expression can be involved in the development and progression of colorectal cancer.

## 2. Materials and Methods

### 2.1. Patients and Collection of Tissue Specimens

Tissue samples were gathered at the Hospital of the Ministry of Internal Affairs and Administration in Olsztyn, Poland, between 2010 and 2013, and 94 patients with confirmed CRC were included in the study (57 men and 37 women; mean age ± standard deviation, 67.85 ± 10.53 years old; ranging from 33 to 91 years). None of the patients had previously undergone radio- or chemotherapy or had a second neoplastic disease. Demographic and clinical data were collected at the time of enrollment. The data on the overall survival time were obtained for all the patients. The median follow-up time was 67.13 months. The control group was composed of 40 healthy subjects (13 men and 27 women; mean age ± standard deviation, 57.11 ± 6.55 years old; ranging from 36 to 74 years) who underwent colonoscopy as part of a routine screening for CRC (the national screening program for early detection of colorectal cancer). Informed consent was obtained from all the subjects involved in the study.

The tissue specimens from the CRC patients were collected during the partial surgical resection of the large intestine, and the specimens from the control group were obtained during the colonoscopies. In a group of CRC patients, the following two kinds of matched samples were collected within 20 min from tumor resection: tumor tissue and macroscopically unaltered, non-cancerous mucosa from the distal portion of the resected large intestine. The tissue specimens were promptly frozen in liquid nitrogen and stored at −80 °C for later use in the quantitative polymerase chain reaction (qPCR) method, whereas the samples for routine histological and immunohistochemical (IHC) evaluation were fixed in 10% neutral-buffered formalin and further processed into paraffin blocks. In the control group of healthy subjects, biopsies were used for routine histological examination and qPCR analysis.

The study was conducted in accordance with the Declaration of Helsinki and was accepted by the Bioethics Committee of the University of Warmia and Mazury in Olsztyn (decisions no. 3/2010, 25 February 2010; 34/2010, 16 December 2010 and 20/2017, 25 May 2017).

### 2.2. Cell Culture

A control cell line derived from normal colon tissue (CCD 841 CoN) and human CRC cell lines with different features (HT-29, SW-480, LoVo) [18] were purchased from the American Type Culture Collection (ATCC; Manassas, VA, USA). The cell lines were maintained in accordance with the supplier’s instructions and were collected at ~80% confluence.

### 2.3. Total RNA Extraction, Reverse Transcription, and Real-Time Quantitative PCR

Total RNA was extracted from the tissue specimens and cell lines tested and reverse transcribed as previously described [19]. The ABI 7500/7500 Fast Real-Time PCR system (Life Technologies, Applied Biosystems, Foster City, CA, USA) was used to quantify *SAV1* gene expression, and the hypoxanthine phosphoribosyltransferase 1 (*HPRT1*) gene was applied as an internal control to normalize the *SAV1* transcript levels. The *SAV1* and *HPRT1* cDNA levels in the gathered samples were assayed using TaqMan^®^ Fast Advanced Master Mix and a respective TaqMan^®^ Gene Expression Assay (for *SAV1*: Hs00560416_m1; and *HPRT1*: Hs02800695_m1; all from Life Technologies, Applied Biosystems) in accordance with the manufacturer’s recommendations. The following conditions were applied: polymerase activation for 20 s at 95 °C, followed by 40 cycles of denaturation at 95 °C for 3 s and annealing/extension at 60 °C for 30 s. The samples underwent amplification in duplicate. For each qPCR run, template-free control reactions were performed. To control the efficiency of the qPCR reactions, standard curves composed of serial dilutions of the corresponding cDNA were used. The ΔΔCt method [20] was applied for relative quantification of *SAV1* expression. The fold-change in the relative gene expression was estimated by calculation of the 2^−ΔΔCt^ value. A fold increase >1 (2^−ΔΔCt^ > 1) indicated an overexpression of *SAV1* in tumor tissue, and a fold decrease <1 (2^−ΔΔCt^ < 1) indicated a downregulation of *SAV1* expression.

### 2.4. Immunohistochemistry and Immunoreactivity Evaluation

SAV1 immunostaining in the CRC and non-cancerous colorectal tissues was performed in accordance with the previously described method [21] using 4 μm thick paraffin sections and the Autostainer Link48 (DakoCytomation, Glostrup, Denmark). Rabbit polyclonal primary antibodies directed against SAV1 (1:50, Anti-SAV1 antibody HPA001808, Sigma-Aldrich, Saint Louis, MO, USA) were applied, while the negative controls were carried out by omitting the primary antibody.

The immunostained SAV1 sections were evaluated using an Olympus BX41 light microscope (Olympus, Tokyo, Japan) by two pathologists in a blinded manner to the patients’ clinicopathological data. The cytoplasmic SAV1 immunoreactivity was estimated according to the Immunoreactive Score (IRS) of Remmele and Stegner [22], taking into account the percentage of cells with a positive reaction (0 points: absence of cells showing positive reaction; 1 point: 1–10%; 2 points: 11–50%; 3 points: 51–80%; and 4 points: over 80% cells showing positive reaction), as well as the reaction intensity (0 points: no reaction; 1 point: low; 2 points: moderate; and 3 points: intense). The final score was the product of both parameters and ranged from 0 to 12 points. The ratio of the scores for cancer cells and epithelial cells of matched non-cancerous tissue was calculated. A ratio value higher than 1 indicated an increase in SAV1 immunoreactivity in the CRC tissue, while a ratio value lower than 1 indicated a decrease.

### 2.5. Database Analysis

*SAV1* RNA-Seq gene expression data of 603 samples derived from primary CRC sites and clinical data of the patients were extracted from the Firehose Broad GDAC (https://gdac.broadinstitute.org/; COADREAD cohort; accessed on 25 October 2023; [23]) and cBioPortal (https://www.cbioportal.org/; TCGA Firehose Legacy dataset; accessed on 17 October 2023 [24]). The patients without *SAV1* expression data were excluded from the analysis. Follow-up data for the overall survival (OS) and disease-free survival (DFS) of the patients were collected for 603 and 525 CRC cases, respectively. Based on the median of the normalized gene expression values of the *SAV1* gene, the entire dataset was divided into two groups (OS and DFS) and subjected to further survival analysis.

### 2.6. Statistical Analysis

All statistical analyses were carried out using STATISTICA 10 (version 10.0; StatSoft, Tulsa, OK, USA) and Prism 6 (version 6.07; GraphPad, La Jolla, CA, USA) software. The differences in *SAV1* mRNA expression levels between the colon mucosa biopsies of healthy individuals and the tissue specimens of CRC patients (tumor and unchanged non-cancerous samples), as well as between the various cell lines, were assessed with the Kruskal–Wallis test followed by Dunn’s multiple comparisons test, whereas the differences in SAV1 protein levels between the matched tumor and non-cancerous specimens of the CRC patients were analyzed using the Wilcoxon matched-pairs test. The associations of *SAV1* expression levels with clinicopathological and demographic parameters were examined with Fisher’s exact test. Survival curves were generated using the Kaplan–Meier method. The statistical significance of differences in survival between groups of patients on the basis of various variables (*SAV1* expression level and clinicopathological and demographic characteristics) was assessed using the log-rank test and the Cox regression method. A *p*-value < 0.05 was considered to indicate a statistically significant difference.

## 3. Results

### 3.1. Downregulated Expression of SAV1 mRNA in CRC Tissues and Cell Lines

**Figure 1:**
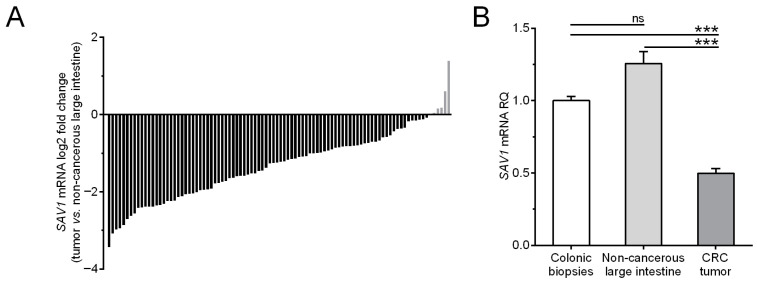
(**A**) The mRNA content of *SAV1* in the colorectal cancer (CRC) tissues of individual patients is presented in relation to the *SAV1* mRNA levels in matched non-cancerous colorectal tissue. (**B**) The average *SAV1* mRNA expression (mean ± SEM) in the tumor and non-cancerous large intestine tissues of CRC patients is presented in relation to the value achieved for the colon mucosa of healthy subjects (1.0). *** *p* < 0.001; ns, differences not statistically significant (*p* > 0.05).

To quantify *SAV1* expression at the mRNA level, the colon biopsies of the healthy individuals and the matched tumor and non-cancerous tissue samples from the CRC patients were subjected to a qPCR analysis. *SAV1* transcripts were detected in all tested colon biopsies of the healthy subjects and tissue specimens of the CRC patients. Among the 94 tumor samples studied, the relative level of *SAV1* mRNA (tumor tissue vs. matched non-cancerous mucosa) was reduced in eighty-eight (93.6%) tumors, while it was elevated in six (6.4%) cases (Table 1, Figure 1A). The average *SAV1* mRNA expression was significantly downregulated in the tumor specimens compared to that observed in the non-cancerous tissue of the CRC patients and the colonic mucosa of the healthy subjects (0.5 ± 0.03 vs. 1.25 ± 0.08 and 1.00 ± 0.03, respectively; *p* < 0.0001; Figure 1B). *SAV1* mRNA levels in the non-cancerous tissues of the CRC patients did not differ significantly from those in the colon biopsies of the healthy individuals (1.25 ± 0.08 vs. 1.00 ± 0.03; *p* > 0.05; Figure 1B). An analysis of TCGA repository data showed slightly lower *SAV1* expression in CRC compared to non-cancerous tissues, but this was not a statistically significant difference (Supplementary Figure S1).

**Figure 2:**
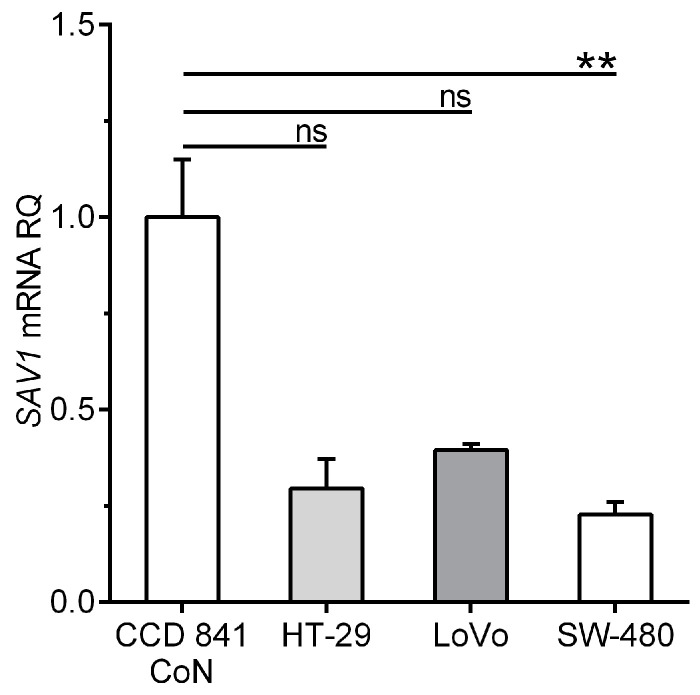
Levels of *SAV1* mRNA expression in the cells of a control colon epithelium-derived cell line (CCD 841 CoN) and selected colorectal cancer (CRC) cell lines (HT-29, LoVo, SW-480). *SAV1* mRNA expression (mean ± SEM) is presented in relation to the value achieved for a control cell line (1.0). The experiment was repeated five times for a control cell line and four times for all CRC cell lines. ** *p* < 0.01; ns, differences not statistically significant (*p* > 0.05).

The qPCR method was also applied to quantify *SAV1* gene expression in a control cell line derived from normal colon tissue and CRC cell lines. The level of *SAV1* mRNA in the cells of the SW-480 line was significantly reduced compared to that in the cells of the CCD 841 CoN line (0.226 ± 0.034 vs. 1.00 ± 0.0087; *p* < 0.05; Figure 2). *SAV1* mRNA expression tended to decrease also in the cells of the other lines, as follows: 0.294 ± 0.077 for HT-29 (about a 3.5-fold decrease; Figure 2) and 0.394 ± 0.015 for LoVo (about a 2.5-fold decrease; Figure 2).

The results obtained with qPCR were used to evaluate the association between *SAV1* mRNA expression and clinicopathological and demographic parameters. Relative *SAV1* mRNA levels were not associated with the parameters studied (sex, age, tumor localization, depth of invasion, presence of metastases, lymph node involvement, and TNM disease stage or grade; *p* > 0.05; Table 1).

### 3.2. Heterogeneous Cytoplasmic Immunoreactivity of SAV1 in CRC Tissues and the Association of Its Decreased Levels with Poorer Clinicopathological Parameters

**Figure 3:**
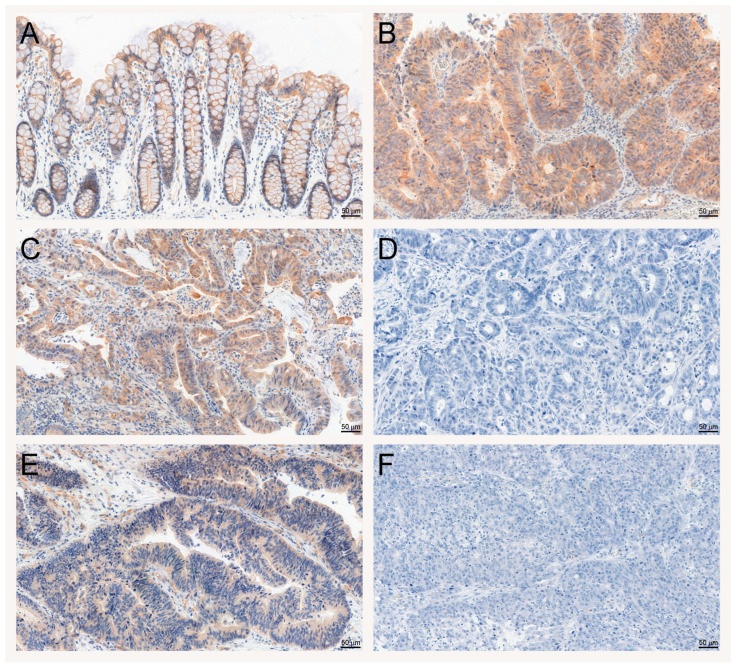
Evaluation of SAV1 protein expression in colorectal cancer (CRC) and non-cancerous colorectal tissues using immunohistochemistry. Immunohistochemical staining of the SAV1 protein in representative non-cancerous (**A**) and tumor (**B**–**F**) tissue specimens of CRC patients. Heterogeneous immunoreactivity of SAV1 depending on lymph node involvement and TNM disease stage—N0, TNM I (**C**) and N2, TNM III (**D**)—and differentiation grade—G2 (**E**) and G3 (**F**). Magnification ×200. Scale bar: 50 µm.

A positive SAV1 immunohistochemical reaction was noted in the cytoplasm of epithelial cells (Figure 3A; Supplementary Figure S2A,C,E,G,I,K,M,O,Q,S; Supplementary Figure S3A,C,E,G,I,K), as well as in the CRC cells of the tissues studied (Figure 3B,C,E; Supplementary Figures S2B,D,F,H,J,R,T and S3B,D,F). Moreover, SAV1 immunostaining was observed in some stromal cells, but they were not accounted in the analysis.

**Figure 4:**
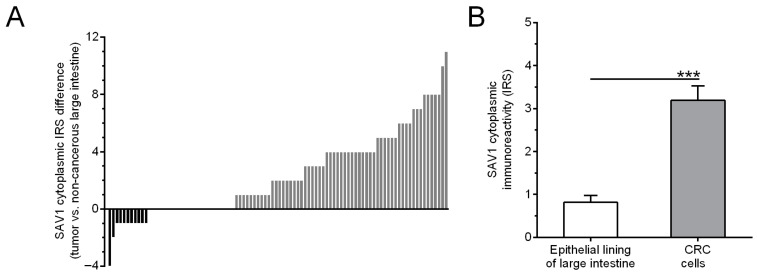
Evaluation of SAV1 protein expression in non-cancerous mucosa and colorectal cancer (CRC) cells using immunohistochemistry. (**A**) The cytoplasmic SAV1 immunoreactivity in the tumors of individual CRC patients is presented in relation to the SAV1 immunoreactivity level in matched non-cancerous colorectal mucosa. (**B**) The average immunoreactivity of the SAV1 protein (mean ± SEM) in epithelial cells of large intestine and colorectal cancer cells. *** *p* < 0.001; IRS—Immunoreactive Score as defined by Remmele and Stegner [22].

SAV1 immunohistochemical staining was observed in tumor cells in 66/94 (70.2%) of the CRC cases studied, whereas, in the epithelial cells of non-cancerous colorectal tissues, SAV1 immunostaining was seen in 43/94 (45.7%) cases. Of the 94 tumor tissue samples studied, the relative immunoreactivity of SAV1 was reduced in 11 (11.7%) and increased in 59 (62.8%) cases, while 24 (25.5%) tumor specimens showed similar levels of SAV1 immunoreactivity to their corresponding non-cancerous tissues cells (Figure 4A). The average score of SAV1 immunostaining was higher in the CRC cells than in the cells of the non-cancerous mucosa (IRS 3.19 ± 0.34 vs. 0.82 ± 0.15, respectively; *p* < 0.0001; Figure 4B).

**Figure 5:**
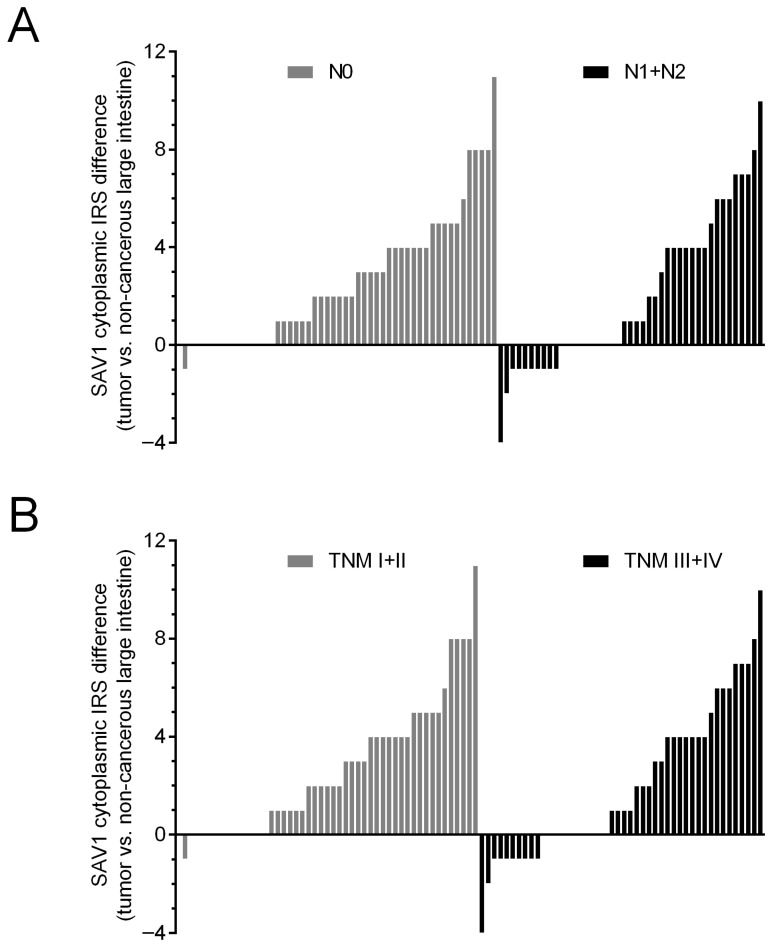
The cytoplasmic SAV1 immunoreactivity in the tumors of individual CRC patients in relation to the SAV1 immunoreactivity level in matched non-cancerous colorectal mucosa depending on N status (**A**) and TNM disease stage (**B**). IRS—Immunoreactive Score [22].

To evaluate the importance of SAV1 protein levels in CRC progression, the association of SAV1 immunoreactivity with selected clinicopathological and demographic features was investigated. The decreased immunoreactivity of SAV1 in the tumor specimens was associated with lymph node involvement (N0 vs. N1 + N2, *p* = 0.0022; Table 1; Figure 3C,D) and higher TNM disease stage (I + II vs. III + IV, *p* = 0.0034; Table 1; Figure 3C,D; Supplementary Figure S2) and histological grade (G2 vs. G3, *p* = 0.0489; Table 1; Figure 3E,F; Supplementary Figure S3). Since we did not observe an association of SAV1 immunoreactivity with M status or a significant difference in SAV1 immunoreactivity between the CRC tissues of the patients with TNM stage III or IV, lymph node involvement appears to be a major contributor to the observed association between SAV1 immunoreactivity and TNM disease stage. Figure 5 presents the cytoplasmic SAV1 immunoreactivity in the tumors of individual CRC patients in relation to the SAV1 immunoreactivity level in matched non-cancerous colorectal mucosa depending on N status (Figure 5A) and TNM stage (Figure 5B).

### 3.3. Overall Patient Survival Is Not Related to the Level of SAV1 Expression in CRC Tissues

**Figure 6:**
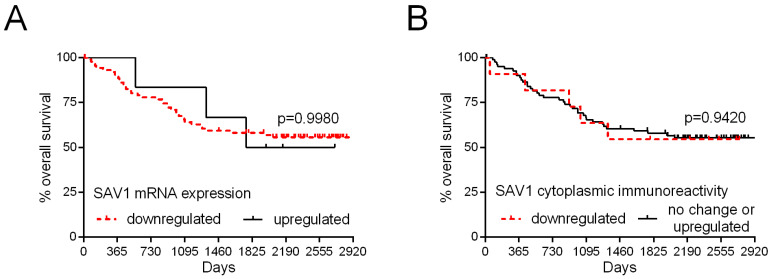
Kaplan–Meier curves of the overall survival of 94 patients with colorectal cancer in relation to *SAV1* mRNA levels (**A**) and SAV1 protein immunoreactivity (**B**).

To estimate the significance of the level of *SAV1* expression as a prognostic factor, all the patients were followed-up on over 67 months. During this period of observation, 41 (43.62%) patients died. The levels of *SAV1* mRNA and SAV1 immunoreactivity were not associated with overall patient survival (Table 2; Figure 6). Of the examined demographic and clinicopathological parameters, the presence of metastases (*p* < 0.0001; Table 2), lymph node involvement (*p* = 0.0006; Table 2), and TNM disease stage (III–IV; *p* < 0.0001; Table 2) were related to poor survival rates. The analysis of TCGA repository data also revealed no association of *SAV1* mRNA expression with the overall patient survival (*p* = 0.5270; Supplementary Figure S4A) and disease-free survival rates (*p* = 0.6529; Supplementary Figure S4B).

## 4. Discussion

CRC is the third most frequently diagnosed cancer in recent years and the second main cause of cancer deaths worldwide. The incidence of CRC is dramatically increasing at an alarming rate. The global number of new cases of CRC is expected to reach 3.2 million in 2040 [25]. Since the symptoms of CRC mainly appear in advanced stages, detecting markers of the early stage of the disease, as well as identifying the mechanisms and factors underlying the development and progression of CRC, are important determinants in the prevention of metastasis, thereby reducing mortality and improving prognosis. A well-established approach in the search for potential cancer biomarkers, as well as prognostic factors and therapeutic targets, is to compare the expression level of genes and their protein products in healthy and cancerous tissues. Since the deactivation of tumor suppressor genes that regulate many cell processes, such as the cell cycle and apoptosis, is often observed in cancer pathogenesis, we decided that the goal of our study would be to analyze the expression level of the *SAV1* gene, which encodes one of the main components of the Hippo suppressor pathway, and to estimate its prognostic significance in CRC.

The importance of the Hippo pathway in gastrointestinal physiology has been confirmed by several studies, and disturbances in the function and expression of its upstream components have been shown to lead to impaired gastrointestinal tissue homeostasis and tumorigenesis [26,27]. In our study, we showed altered expression of the *SAV1* gene in tumors compared to healthy and non-cancerous colon tissues. Our observations of significantly lower levels of *SAV1* mRNA in CRC tissues are consistent with the findings of Jiang et al. [16,17], who, however, used tissues collected from a much smaller number of patients (*n* = 12 and *n* = 20), and Sun et al. [28] (*n* = 46). Moreover, we used an additional control in the form of tissues from routine colon biopsies of healthy subjects, in addition to non-cancerous tissues from CRC patients, to compare the *SAV1* mRNA expression levels. To explain our results, one should take into account the role of various types of microRNAs (miRNAs) that have been previously shown to control the expression of *SAV1*, such as miR-21 [17], miR-590-3p [28], and miR-103a-3p [29], small oligonucleotides which can inhibit the expression of the target mRNA in post-transcriptional gene regulatory pathways. Namely, increased levels of the above-mentioned miRNAs in CRC tissues and a negative correlation with *SAV1* mRNA expression were found in some studies [17,28,29], which is in line with the results of our study. We also showed a reduction in *SAV1* mRNA expression in CRC cell lines. There has been no comparison of *SAV1* expression at the mRNA level in CRC and control cell lines in previous studies, but Jiang et al. [17] demonstrated decreased levels of the SAV1 protein in CRC cell lines. Studies conducted by the aforementioned group also revealed that *SAV1* knockdown promoted the growth of CRC cells in vitro and in vivo [16]. Also, Cai et al. [12] reported that *SAV1* knockout mice developed colonic polyps, which resembled a human lesion named sessile serrated polyps, suggesting a role for altered *SAV1* expression in the development of CRC. In light of these studies, it was surprising for us to observe higher average SAV1 immunostaining in tumor tissues compared to non-cancerous tissues. A previous study by Jiang et al. [16], using a Western blot method and tissues from 12 patients, revealed reduced levels of the SAV1 protein in CRC tissues. Part of the observed discrepancy in the protein expression profile may be due to the fact that, in the Western blot method, the protein level is determined in the tissue homogenate, while the IHC method we used allows for the precise localization of the protein, and we determined the level of SAV1 immunoreactivity only in epithelial or CRC cells. Another reason could be the highly complex epigenetic and post-transcriptional regulation of *SAV1* gene expression, which could also explain the differences in mRNA and protein expression levels. Won et al. [30] discovered that an anti-apoptotic protein BCL-2 could stimulate the proteasomal degradation of the SAV1 protein through direct binding to SAV1 and that BCL-2 overexpression did not lead to a reduction in *SAV1* mRNA levels, indicating that this phenomenon occurs at a post-transcriptional level. Moreover, they demonstrated in MCF7 (human breast adenocarcinoma) cells high levels of the SAV1 protein but low levels of the BCL-2 protein. By contrast, cells of other cell lines such as SH-SY5Y (human neuroblastoma) had very low levels of the SAV1 protein but very high protein levels of BCL-2. The inverse correlation of SAV1 and BCL-2 expression, as well as the fact that BCL-2 expression was found to be decreased in CRC [31], may, to some extent, explain the elevated average SAV1 protein immunoreactivity we observed in CRC.

Although Wang et al. [32] demonstrated that *SAV1* was silenced by DNA methylation in the AsPC-1 and SW1990 pancreatic cancer cell lines, many other studies have shown that the regulation of *SAV1* expression by DNA methylation or mutation is not common in cancer [8,33,34,35,36,37]. Some authors suggested that MST2-mediated phosphorylation of the SAV1 protein stabilizes this protein and increases the interaction of SAV1 with MST2, which, in turn, increases the levels of these proteins. However, it was also showed that SAV1 phosphorylation may directly inhibit ubiquitin ligases and proteasome-mediated degradation and, thus, increase SAV1 levels [38,39]. Undoubtedly, the mechanisms regulating the SAV1 protein level require further investigations, especially since reports on SAV1 protein expression in various cancers are inconsistent.

In line with our findings, Xu et al. [40] noted that SAV1 immunoreactivity tended to increase from normal mucosa, through intestinal metaplasia, to gastric cancer. On the other hand, Wang et al. [13] reported lower SAV1 immunostaining in pancreatic ductal adenocarcinoma than in paratumor tissues. Similarly, Matsuura et al. [15] found that SAV1 protein expression was downregulated in clear cell renal cell carcinoma samples. Li et al. [41] also demonstrated that the SAV1 protein level was decreased in lung cancer tissue. Altogether, these findings suggest that SAV1 may be expressed in a cancer-type-specific manner. A study by Lee et al. [11], who created conditional knockout mice in which the *SAV1* gene was inactivated specifically in the liver, indicated that SAV1 played a minor role in differentiated hepatocytes and in the maintenance of their quiescence. Hepatocytes of the liver-specific SAV1 knockout mice presented only marginal, if any, upregulation of YAP and a proliferative index comparable to that of the control hepatocytes. In contrast, the accumulation of YAP and other Hippo pathway components in the oval cells (hepatic progenitor cells) of the SAV1-deficient liver was observed. The proliferation index of these oval cells was elevated. The liver-specific SAV1 knockout mice developed tumors, thought to originate from the transformed oval cells, with a mixed (hepatocellular carcinoma/cholangiocarcinoma) phenotype, distinct from hepatocellular carcinoma, originating from the aberrant proliferation of hepatocytes [11]. Accurately assessing the role of impaired SAV1 expression in tumorigenesis requires an understanding of the broad spectrum of factors that collaborate with SAV1. Kim et al. [42] demonstrated that SAV1-knockout or 15-PDGH (hydroxyprostaglandin dehydrogenase 15)-knockout mice did not develop spontaneous tumors after the induction of colitis, but the SAV1/15-PDGH double knockout mice developed polyps which eventually progressed to carcinoma in situ, suggesting a cooperation of the loss of both in cancer development.

To the best of our knowledge, the association of *SAV1* expression in tumor samples with the clinicopathological parameters of CRC patients, as well as their overall survival, has not yet been reported. Although our study showed that the average SAV1 immunoreactivity was increased in the CRC tissue compared to the non-cancerous large intestine tissue, we observed a reduced SAV1 protein immunoreactivity in the tumor tissues from the patients with worse clinicopathological parameters. A decreased SAV1 protein immunoreactivity in the tumor samples was associated with lymph node involvement and higher TNM disease stage and histological grade, confirming the tumor-suppressive role of SAV1. Our findings are consistent with those obtained for several other types of cancers. Xu et al. [40] noted that SAV1 immunoreactivity was higher in gastric cancer without lymph node metastasis compared to metastatic cancer. Immunohistochemical analyses of pancreatic ductal adenocarcinoma showed that a low expression of the SAV1 protein was related to unfavorable clinicopathological features, such as histological differentiation and lymph node status [13]. Matsuura et al. [15] found that SAV1 downregulation at the protein level was correlated with tumor grade occurring preferentially in high-grade clear cell renal cell carcinoma. An in silico analysis by de Amorim et al. [14] indicated *SAV1* upregulation at the mRNA and protein levels in the early stages of breast cancer. In our study, we observed no significant association between the *SAV1* mRNA expression levels and the clinicopathological features of the CRC patients, and the *SAV1* mRNA and protein levels were not significantly correlated with the patients’ overall survival. In contrast, *SAV1* mRNA upregulation in breast cancer was associated with a better survival probability [14], and the elevated expression of the SAV1 protein in pancreatic ductal adenocarcinoma was a significant favorable prognostic factor of overall survival [13].

## 5. Conclusions

The *SAV1* mRNA and protein levels in the CRC tissues were significantly different from those noted in the non-cancerous mucosa, and it can, therefore, be assumed that the impaired expression of *SAV1* is associated with the development of CRC. We are the first to provide evidence that altered SAV1 protein expression is related to unfavorable clinicopathological parameters in CRC patients, such as lymph node involvement and higher TNM disease stage and histological grade, and, thus, appears to be involved in CRC progression. A better understanding of the mechanisms controlling *SAV1* expression and those leading to its alterations can provide deeper insights into the pathogenesis of CRC.

## Figures and Tables

**Table 1:** Associations between the relative expression of *SAV1* mRNA or SAV1 immunoreactivity and clinicopathological and demographic features of the patients with colorectal cancer (CRC).

Parameters	N	*SAV1* mRNA RQ (CRC vs. Non-Cancerous Large Intestine)			SAV1 Cytoplasmic IRS (CRC vs. Non-Cancerous Large Intestine)		
		RQ < 1 n (%)	RQ > 1 n (%)	*p*-Value	Downregulated n (%)	Upregulated or No Change n (%)	*p*-Value
Total	94	88 (93.6)	6 (6.4)		11 (11.7)	83 (88.3)	
Sex							
Men	57	53 (93.0)	4 (7.0)	1.0000	7 (12.3)	50 (87.7)	1.0000
Women	37	35 (94.6)	2 (5.4)		4 (10.8)	33 (89.2)	
Age (median = 67.5)							
<67.5 years	47	42 (89.4)	5 (10.6)	0.2035	8 (17.0)	39 (83.0)	0.1979
>67.5 years	47	46 (97.9)	1 (2.1)		3 (6.4)	44 (93.6)	
Localization							
Cecum and ascending colon	33	30 (90.9)	3 (9.1)	0.7184	6 (18.2)	27 (81.8)	0.3558
Transverse, descending, and sigmoid colon	24	23 (95.8)	1 (4.2)		2 (8.3)	22 (91.7)	
Rectum	37	35 (94.6)	2 (5.4)		3 (8.1)	34 (91.9)	
Depth of invasion (T status)							
T1 + T2	14	13 (92.9)	1 (7.1)	1.0000	1 (7.1)	13 (92.9)	1.0000
T3 + T4	80	75 (93.8)	5 (6.2)		10 (12.5)	70 (87.5)	
Lymph node metastasis (N status)							
N0	51	48 (94.1)	3 (5.9)	1.0000	1 (2.0)	50 (98.0)	**0.0022**
N1 + N2	43	40 (93.0)	3 (7.0)		10 (23.3)	33 (76.7)	
Metastasis (M status)							
M0	83	77 (92.8)	6 (7.2)	1.0000	11 (13.3)	72 (86.7)	0.3506
M1	11	11 (100.0)	0 (0.0)		0 (0.0)	11 (100.0)	
TNM stage							
I + II	48	45 (93.8)	3 (6.2)	1.0000	1 (2.1)	47 (97.9)	**0.0034**
III + IV	46	43 (93.5)	3 (6.5)		10 (21.7)	36 (78.3)	
Grade of differentiation							
G2	86	81 (94.2)	5 (5.8)	0.4226	8 (9.3)	78 (90.7)	**0.0489**
G3	8	7 (87.5)	1 (12.5)		3 (37.5)	5 (62.5)	

Significant *p*-values (*p* < 0.05) are in bold. RQ—relative quantification; IRS—Immunoreactive Score of Remmele and Stegner.

**Table 2:** Univariate and multivariate Cox regression analyses of the overall survival rates related to different prognostic variables in patients with colorectal cancer.

Parameters	Overall Survival—Cox Regression			
	Univariate		Multivariate	
	HR (95% CI)	*p*-Value	HR (95% CI)	*p*-Value
*SAV1* mRNA RQ (downregulated vs. upregulated)	1.01 (0.31–3.27)	0.9880		
SAV1 IRS (downregulated vs. no change/upregulated)	1.03 (0.41–2.64)	0.9412		
Sex (women vs. men)	0.72 (0.38–1.37)	0.3120		
Age (>67.5 vs. <67.5 years)	1.55 (0.84–2.89)	0.1593		
Localization				
(rectum vs. transverse, descending, and sigmoid colon)	1.15 (0.54–2.46)	0.4798		
(rectum vs. cecum and ascending colon)	0.80 (0.39–1.65)	0.3924		
Depth of invasion (T status) (T3 + T4 vs. T1 + T2)	2.82 (0.87–9.15)	0.0834		
Lymph node metastasis (N status) (N1 + N2 vs. N0)	3.12 (1.63–5.98)	**0.0006**	0.99 (0.26–3.85)	0.9919
Metastasis (M status) (M1 vs. M0)	6.39 (3.00–13.62)	**<0.0001**	3.68 (1.51–8.96)	**0.0041**
TNM stage (III + IV vs. I + II)	4.17 (2.08–8.37)	**<0.0001**	3.27 (0.70–15.34)	0.1322
Grade of differentiation (G3 vs. G2)	0.85 (0.26–2.74)	0.7793		

Significant *p*-values (*p* < 0.05) are in bold. HR—hazard ratio; CI—confidence interval; RQ—relative quantification; IRS—Immunoreactive Score of Remmele and Stegner.

## Data Availability

The data were collected from the Firehose Broad GDAC (https://gdac.broadinstitute.org/; COADREAD cohort; accessed on 25 October 2023) and cBioPortal (https://www.cbioportal.org/; TCGA Firehose Legacy dataset; accessed on 17 October 2023).

## Supplementary Materials

The following supporting information can be downloaded at https://www.mdpi.com/article/10.3390/cancers15245771/s1: Figure S1: *SAV1* mRNA expression in colorectal cancer (CRC) and non-cancerous large intestine samples from the TCGA repository database (COADREAD dataset); Figure S2: Evaluation of SAV1 protein expression in colorectal cancer (CRC) and non-cancerous colorectal tissues using immunohistochemistry. Heterogeneous immunoreactivity of SAV1 depending on TNM disease stage; Figure S3: Evaluation of SAV1 protein expression in colorectal cancer (CRC) and non-cancerous colorectal tissues using immunohistochemistry. Heterogeneous immunoreactivity of SAV1 depending on differentiation grade; Figure S4: Kaplan–Meier curves of overall survival (A) of 603 patients and disease-free survival (B) of 525 patients with colorectal cancer in relation to *SAV1 mRNA* levels on the basis of the database in the TCGA repository (COADREAD dataset).

## References

[1] Sebio A., Lenz H.J. (2015). Molecular Pathways: Hippo Signaling, a Critical Tumor Suppressor. Clin. Cancer Res..

[2] Mo J.S., Park H.W., Guan K.L. (2014). The Hippo signaling pathway in stem cell biology and cancer. EMBO Rep..

[3] Pan D. (2010). The hippo signaling pathway in development and cancer. Dev. Cell..

[4] Bao Y., Hata Y., Ikeda M., Withanage K. (2011). Mammalian Hippo pathway: From development to cancer and beyond. J. Biochem..

[5] Liang K., Zhou G., Zhang Q., Li J., Zhang C. (2014). Expression of hippo pathway in colorectal cancer. Saudi J. Gastroenterol..

[6] Wierzbicki P.M., Rybarczyk A. (2015). The Hippo pathway in colorectal cancer. Folia Histochem. Cytobiol..

[7] Sung H., Ferlay J., Siegel R.L., Laversanne M., Soerjomataram I., Jemal A., Bray F. (2021). Global cancer statistics 2020: GLOBOCAN estimates of incidence and mortality worldwide for 36 cancers in 185 countries. CA Cancer J Clin..

[8] de Amorim Í.S.S., de Sousa Rodrigues M.M., Mencalha A.L. (2021). The tumor suppressor role of salvador family WW domain-containing protein 1 (SAV1): One of the key pieces of the tumor puzzle. J. Cancer Res. Clin. Oncol..

[9] Donninger H., Allen N., Henson A., Pogue J., Williams A., Gordon L., Kassler S., Dunwell T., Latif F., Clark G.J. (2011). Salvador protein is a tumor suppressor effector of RASSF1A with hippo pathway-independent functions. J. Biol. Chem..

[10] Lee J.H., Kim T.S., Yang T.H., Koo B.K., Oh S.P., Lee K.P., Oh H.J., Lee S.H., Kong Y.Y., Kim J.M. (2008). A crucial role of WW45 in developing epithelial tissues in the mouse. EMBO J..

[11] Lee K.P., Lee J.H., Kim T.S., Kim T.H., Park H.D., Byun J.S., Kim M.C., Jeong W.I., Calvisi D.F., Kim J.M. (2010). The Hippo-Salvador pathway restrains hepatic oval cell proliferation, liver size, and liver tumorigenesis. Proc. Natl. Acad. Sci. USA.

[12] Cai J., Zhang N., Zheng Y., de Wilde R.F., Maitra A., Pan D. (2010). The Hippo signaling pathway restricts the oncogenic potential of an intestinal regeneration program. Genes. Dev..

[13] Wang L., Wang Y., Li P.P., Wang R., Zhu Y., Zheng F., Li L., Cui J.J., Wang L.W. (2016). Expression profile and prognostic value of SAV1 in patients with pancreatic ductal adenocarcinoma. Tumour Biol..

[14] de Amorim Í.S.S., Dias I.X., Pinheiro D., de Carvalho S.N., Nicolau-Neto P., Rodrigues J.A., Siqueira P.B., Oliveira M.D.S., Panis C., da Fonseca A.S. (2023). Profiles of Expression of SAV1 in Normoxia or Hypoxia Microenviroment are Associated with Breast Cancer Prognosis. Arch. Med. Res..

[15] Matsuura K., Nakada C., Mashio M., Narimatsu T., Yoshimoto T., Tanigawa M., Tsukamoto Y., Hijiya N., Takeuchi I., Nomura T. (2011). Downregulation of SAV1 plays a role in pathogenesis of high-grade clear cell renal cell carcinoma. BMC Cancer.

[16] Jiang J., Chang W., Fu Y., Gao Y., Zhao C., Zhang X., Zhang S. (2017). SAV1 represses the development of human colorectal cancer by regulating the Akt-mTOR pathway in a YAP-dependent manner. Cell Prolif..

[17] Jiang J., Chang W., Fu Y., Gao Y., Zhao C., Zhang X., Zhang S. (2019). SAV1, regulated by microRNA-21, suppresses tumor growth in colorectal cancer. Biochem. Cell Biol..

[18] Flatmark K., Maelandsmo G.M., Martinsen M., Rasmussen H., Fodstad Ø. (2004). Twelve colorectal cancer cell lines exhibit highly variable growth and metastatic capacities in an orthotopic model in nude mice. Eur. J. Cancer.

[19] Kowalczyk A.E., Krazinski B.E., Godlewski J., Kiewisz J., Kwiatkowski P., Sliwinska-Jewsiewicka A., Kiezun J., Wierzbicki P.M., Bodek G., Sulik M. (2015). Altered expression of the *PLAGL1* (*ZAC1/LOT1*) gene in colorectal cancer: Correlations to the clinicopathological parameters. Int. J. Oncol..

[20] Livak K.J., Schmittgen T.D. (2001). Analysis of relative gene expression data using real-time quantitative PCR and the 2-ΔΔCT method. Methods.

[21] Kowalczyk A.E., Godlewski J., Krazinski B.E., Kiewisz J., Sliwinska-Jewsiewicka A., Kwiatkowski P., Pula B., Dziegiel P., Janiszewski J., Wierzbicki P.M. (2015). Divergent expression patterns of SATB1 mRNA and SATB1 protein in colorectal cancer and normal tissues. Tumour. Biol..

[22] Remmele W., Stegner H.E. (1987). Recommendation for uniform definition of an immunoreactive score (IRS) for immunohistochemical estrogen receptor detection (ER-ICA) in breast cancer. Pathologe.

[23] Broad Institute TCGA Genome Data Analysis Center (2016). Analysis Overview for Colorectal Adenocarcinoma (Primary Solid Tumor Cohort)—28 January 2016.

[24] Cerami E., Gao J., Dogrusoz U., Gross B.E., Sumer S.O., Aksoy A., Jacobsen A., Byrne C.J., Heuer M.L., Larsson E. (2012). The CBio Cancer Genomics Portal: An Open Platform for Exploring Multidimensional Cancer Genomics Data. Cancer Discov..

[25] Xi Y., Xu P. (2021). Global colorectal cancer burden in 2020 and projections to 2040. Transl Oncol..

[26] Yu F.X., Meng Z., Plouffe S.W., Guan K.L. (2015). Hippo pathway regulation of gastrointestinal tissues. Annu. Rev. Physiol..

[27] Hong A.W., Meng Z., Guan K.L. (2016). The Hippo pathway in intestinal regeneration and disease. Nat. Rev. Gastroenterol. Hepatol..

[28] Sun Z.Q., Shi K., Zhou Q.B., Zeng X.Y., Liu J., Yang S.X., Wang Q.S., Li Z., Wang G.X., Song J.M. (2017). MiR-590-3p promotes proliferation and metastasis of colorectal cancer via Hippo pathway. Oncotarget.

[29] Sun Z., Zhang Q., Yuan W., Li X., Chen C., Guo Y., Shao B., Dang Q., Zhou Q., Wang Q. (2020). MiR-103a-3p promotes tumour glycolysis in colorectal cancer via hippo/YAP1/HIF1A axis. J. Exp. Clin. Cancer Res..

[30] Won G.W., Park S.H., Park J., Lee Y., Lee Y.H. (2019). Mammalian Hippo kinase pathway is downregulated by BCL-2 via protein degradation. Biochem. Biophys. Res. Commun..

[31] Ramesh P., Medema J.P. (2020). BCL-2 family deregulation in colorectal cancer: Potential for BH3 mimetics in therapy. Apoptosis.

[32] Wang L., Wang M., Hu C., Li P., Qiao Y., Xia Y., Liu L., Jiang X. (2017). Protein salvador homolog 1 acts as a tumor suppressor and is modulated by hypermethylation in pancreatic ductal adenocarcinoma. Oncotarget.

[33] Hill V.K., Dunwell T.L., Catchpoole D., Krex D., Brini A.T., Griffiths M., Craddock C., Maher E.R., Latif F. (2011). Frequent epigenetic inactivation of KIBRA, an upstream member of the Salvador/Warts/Hippo (SWH) tumor suppressor network, is associated with specific genetic event in B-cell acute lymphocytic leukemia. Epigenetics.

[34] Seidel C., Schagdarsurengin U., Blümke K., Würl P., Pfeifer G.P., Hauptmann S., Taubert H., Dammann R. (2007). Frequent hypermethylation of MST1 and MST2 in soft tissue sarcoma. Mol. Carcinog..

[35] Zhang X., Guo C., Wu X., Li A.X., Liu L., Tsark W., Dammann R., Shen H., Vonderfecht S.L., Pfeifer G.P. (2016). Analysis of Liver Tumor-Prone Mouse Models of the Hippo Kinase Scaffold Proteins RASSF1A and SAV1. Cancer Res..

[36] Yoo N.J., Soung Y.H., Lee J.W., Park W.S., Kim S.Y., Nam S.W., Han J.H., Kim S.H., Lee J.Y., Lee S.H. (2003). Mutational analysis of salvador gene in human carcinomas. APMIS.

[37] Mehra R., Vats P., Cieslik M., Cao X., Su F., Shukla S., Udager A.M., Wang R., Pan J., Kasaian K. (2016). Biallelic Alteration and Dysregulation of the Hippo Pathway in Mucinous Tubular and Spindle Cell Carcinoma of the Kidney. Cancer Discov..

[38] Park B.H., Lee Y.H. (2011). Phosphorylation of SAV1 by mammalian ste20-like kinase promotes cell death. BMB Rep..

[39] Callus B.A., Verhagen A.M., Vaux D.L. (2006). Association of mammalian sterile twenty kinases, Mst1 and Mst2, with hSalvador via C-terminal coiled-coil domains, leads to its stabilization and phosphorylation. FEBS J..

[40] Xu Z.P., Zhu J.S., Zhang Q., Wang X.Y. (2011). A breakdown of the Hippo pathway in gastric cancer. Hepatogastroenterology.

[41] Li X., Zhou X., Fan Y., Zhang Y., Zu L., Yao F., Zhou Q. (2016). WW45, a Gli1 binding protein, negatively regulated Hedgehog signaling in lung cancer. Oncotarget.

[42] Kim H.B., Kim M., Park Y.S., Park I., Kim T., Yang S.Y., Cho C.J., Hwang D., Jung J.H., Markowitz S.D. (2017). Prostaglandin E2 Activates YAP and a Positive-Signaling Loop to Promote Colon Regeneration After Colitis but Also Carcinogenesis in Mice. Gastroenterology.

