# Supplementary material for: Impaired Expression of the Salvador Homolog-1 Gene Is Associated with the Development and Progression of Colorectal Cancer
Source: Cancers (Basel). 2023 Dec 8;15(24):5771. doi: 10.3390/cancers15245771 (PMC10742029; doi:10.3390/cancers15245771)
Supplement: Supplementary file 1 [file cancers-15-05771-s001.zip › Supplementary Figure S2.pdf]

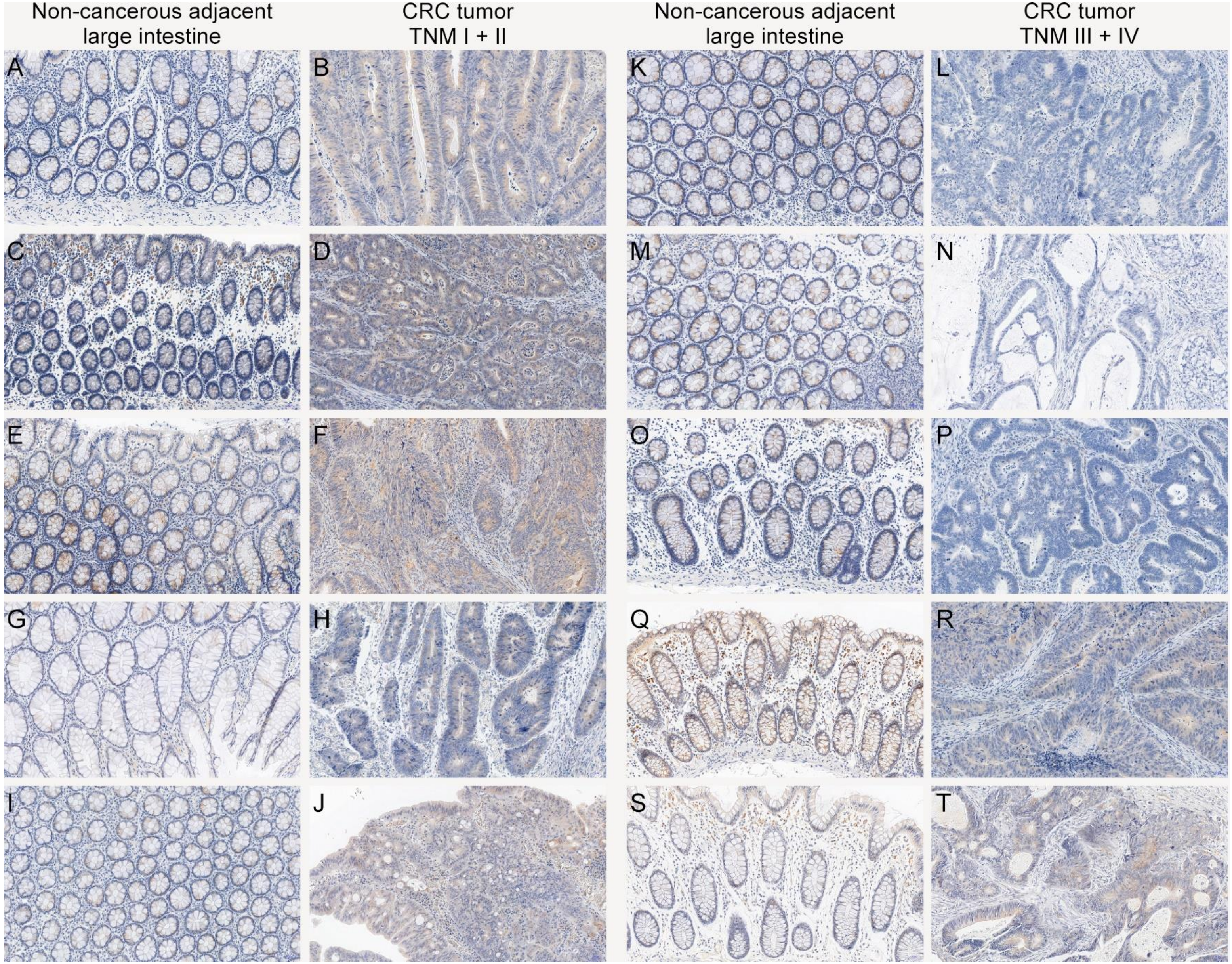

Figure S2. Evaluation of SAV1 protein expression in colorectal cancer (CRC) and non-cancerous colorectal tissues using immunohistochemistry. Heterogeneous immunoreactivity of SAV1 depending on TNM disease stage. Magnification ×200
