# Supplementary material for: Impaired Expression of the Salvador Homolog-1 Gene Is Associated with the Development and Progression of Colorectal Cancer
Source: Cancers (Basel). 2023 Dec 8;15(24):5771. doi: 10.3390/cancers15245771 (PMC10742029; doi:10.3390/cancers15245771)
Supplement: Supplementary file 1 [file cancers-15-05771-s001.zip › Supplementary Figures S1 and S4.pdf]

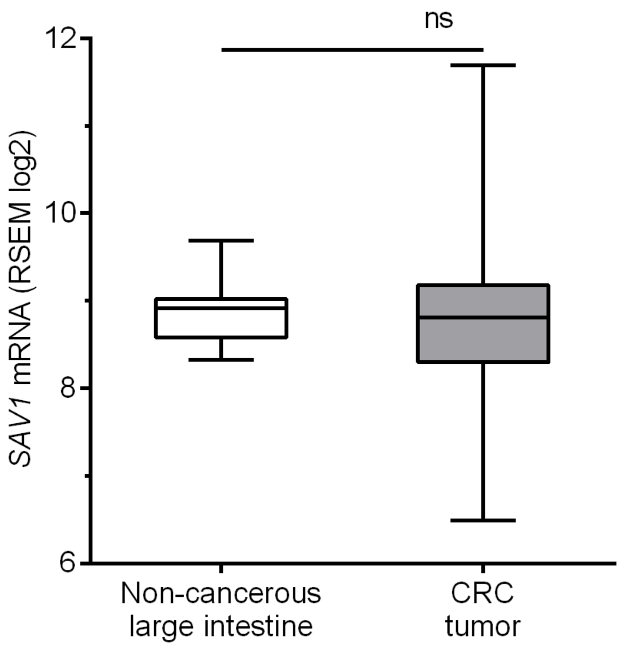

Figure S1. *SAV1* mRNA expression in colorectal cancer (CRC) and non-cancerous large intestine samples from the TCGA repository database (COADREAD dataset). Data are presented as box-plots (median with 25th and 75th percentile and the whiskers showing minimum and maximum). ns, differences not statistically significant (p>0.05).

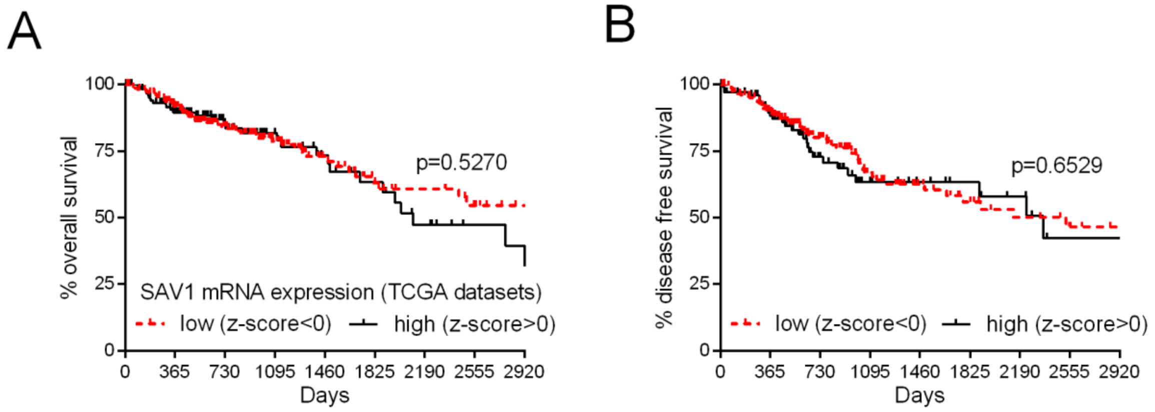

Figure S4. Kaplan-Meier curves of overall survival (**A**) of 603 patients and disease-free survival (**B**) of 525 patients with colorectal cancer in relation to *SAV1* mRNA levels on the basis of the database in the TCGA repository (COADREAD dataset).
